# Fall Seven Times, Stand Up Eight: Linking Project Management Innovation, Project Governance, and High-Performance Work Practices to Project Success

**DOI:** 10.3389/fpsyg.2022.902816

**Published:** 2022-05-17

**Authors:** Umer Zaman, Mohammad Nasar Khan, Syed Hassan Raza, Pablo Farías

**Affiliations:** ^1^Endicott College of International Studies (ECIS), Woosong University, Daejeon, South Korea; ^2^Department of Management Sciences, Shaheed Zulfikar Ali Bhutto Institute of Science and Technology (SZABIST), Islamabad, Pakistan; ^3^Department of Communication Studies, Bahauddin Zakariya University, Multan, Pakistan; ^4^Departamento de Administración, Facultad de Economía y Negocios, Universidad de Chile, Santiago, Chile

**Keywords:** project management innovation, project governance, high-performance work practices, project success, structural equation modeling

## Abstract

Project managers seem to be puzzled in resolving the global dilemma of project failures across industries. Hence, the present study introduces project management innovation (PMI) as a determinant of project success (PS) and explores whether project governance (PG) and high-performance work practices (HPWPs), strengthen this relationship. To confirm these propositions, study data using adapted scales were collected from project professionals representing software development companies in the emerging IT industry in Pakistan. Structural equation modeling (SEM) was employed to examine the hypothesized relationships and encourage PMI-guided solutions for project failures. SEM results statistically validated that project success is positively influenced by PMI, whereas this relationship is significantly strengthened through the moderating influence of PG and HPWPs, respectively. Theoretically, the present research is the first of its kind to introduce and empirically examine these untested relationships between PMI, PG, HPWPs, and PS in a single framework. These novel findings hold strategic value for both project managers and organizational leaders who oversee a range of project portfolios. Long-lasting advantages and superior achievements can be reinvigorated through PMI, after departure from traditional approaches and answering calls for new solutions to new problems in managing projects. Moreover, project governance and HPWPs should be reconfigured to oversee, as well as meet the special needs of each unique project.

## Introduction

Despite a series of scientific contributions to project management literature in the recent decade (Zaman et al., 2022c), the global project management industry has experienced an astonishing rate of project failures (Gartner Inc, 2013; KPMG, 2017; PMI, 2018; Standish Group Report, 2018). Annually, project management companies lose the United States $99 million for every United States $1 trillion invested in projects (PMI, 2018; Zaman et al., 2022b). Nonetheless, project success has continued to receive significant attention in major studies that introduce various elements in the project success framework (Khan and Rasheed, 2015; Aga et al., 2016; Musawir et al., 2017; Wu et al., 2017; Zaman et al., 2022c). The increasing attempts to fill gaps in project management research that bring improvements to how projects are managed today (Zaman et al., 2021a); still, there has never been an examination of project success from the perspective of project management innovation (Musawir et al., 2017; Zaman et al., 2022c). There is widespread international evidence that persistently supports the dynamic capabilities of management innovation in achieving successful organizational outcomes (Millar et al., 2018; Hassi, 2019; Khosravi et al., 2019). As project management organizations continue to strive for better operations (rather than innovating) in the management of their projects, this undermines the strategic value of project management hence showing a staggering rate of 50% of project failures (PMI, 2018; Zaman et al., 2022b). Modern projects are exposed to extremely volatile and unforgiving project environments that require project managers to be highly adaptive and resilient, while also remaining focused on efficient ways to meet their project goals (Bryde et al., 2018; Zaman et al., 2022b). However, project management researchers and practitioners have struggled to codify the best project management practices, rather than looking beyond it (Zaman et al., 2022c).

The present study argues that project management innovation should be no less momentous than the spawned changes in the modern-project management environment (Millar et al., 2018; Zaman et al., 2022c). Project managers must adapt to the innovation-infused project management in order to meet imperative performance needs (Millar et al., 2018). The present study provides emboldened thoughts to academics and project practitioners to spur the renewal of the traditional project management approaches. As modern projects continue to face frequently unexpected risks with different levels of predictability and impact (Zaman et al., 2021b), this makes it critical for project managers to be aggressive adopters by using project management innovation as their survival toolkit for future projects (Khosravi et al., 2019; Zaman et al., 2022c). Managerial innovation has embraced significant recognition due to its critical role in organizational renewal, creativity, performance, competitive advantage, and organizational long-term sustainable success (Hassi, 2019; Khosravi et al., 2019). Management innovation has been showcased as a way of life in the VUCA world, i.e., volatility, uncertainty, complexity, and ambiguity (Millar et al., 2018; Zaman et al., 2020).

Management innovation is a non-technological term that has not long appeared in management research and currently remains an under-studied topic. Unlike technological innovations, non-technological ones’ (e.g., management innovation) are highly difficult and challenging to be replicated owing to its organization-oriented nature (Hassi, 2019; Zaman et al., 2020). Hence, management innovation enables organizations to achieve sustainable competitive advantage and high competitiveness due to its radical and systemic nature (Hassi, 2019). Management innovation signifies the managers’ capabilities to stimulate innovation within firms and it attracts novel managerial structures, processes, and practices for the adopting entity. Total quality management (TQM), just-in-time (JIT) production, quality circles, and 360-degree response are some instances of management innovation (Hassi, 2019; Khosravi et al., 2019). A latest longitudinal study analysis by Berggren (2019) has raised the need for ambidextrous project management by way of bridging two streams of literature and mutual recognition of innovation and project management research (Zaman et al., 2020).

Moreover, there has been a growing corpus of studies on project governance, as it may potentially emerge as a mainstream domain of project management research, theory, and practice (Musawir et al., 2017; Riis et al., 2019). Project governance mechanism provides a rationalized basis for allocating project resources through effectively controlled and coordinated project activities within project structures, systems, and processes (Musawir et al., 2017). A meta-analysis of research established the importance of project governance in ensuring successful project delivery and ultimately realizing project success (Joslin and Müller, 2016; Musawir et al., 2017; Brunet, 2019). Project governance mechanism also resolves conflicting issues during the interface between managing projects and managing parent organizations. Project governance has shown diversified awareness of its increasing project applications, which may vary in projects, programs, and project portfolios (Müller et al., 2015). Project governance displays robust actions in reducing project transaction costs besides endorsing project performance (Cardenas et al., 2017). Similarly, high-performance work practices have shown widely documented support for organizations in establishing high levels of performance outcomes (Zaman, 2020). High-performance work practices include a broad range of bundled innovative practices and processes that demonstrate mutually reinforcing and synergistic impact on employee and organizational level outcomes (Úbeda-García et al., 2018; Zaman, 2020). However, the degree of implementation of high-performance work practices varies across industries and organizations. HPWP-derived benefits may not be realized due to its implementation inefficiencies. Moreover, high-performance work practices may also reap differential benefits for a variety of organizational contexts, including firm size, reputation, industry, workforce composition, business, and HR strategies (Stirpe and Zárraga-Oberty, 2017; Zaman, 2020).

Despite growing recognition of project governance and high-performance work practices in promising opportunities for project success (Khan and Rasheed, 2015; Musawir et al., 2017), there is rare evidence of the combined effects of project governance and high-performance work practices in a single framework of project success. Following an examination of scientific literature, the present study developed an inclusive and novel framework of project success in the software industry involving the interaction effects of project governance and high-performance work practices on the association between project management innovation and project success. Thereby, this research aims to answer two research questions. First, what is the effect of project management innovation on project success? Second, do project governance and high-performance work practices moderate the effects of project management innovation on project success?

Prior research has only been able to scratch the surface while examining project success from the perspective of project management innovation. Hence, this research has two-fold implications from a managerial standpoint. First, project management organizations need to gain from the tremendous advantages of innovation-infused project management to secure greater project success (Hassi, 2019; Khosravi et al., 2019; Zaman et al., 2020). Second, the relationship between project management innovation and project success could be further intensified through the interaction of project governance and high-performance work practices in project-based environments (Khan and Rasheed, 2015; Musawir et al., 2017).

The remainder of this paper is arranged as follows. The next section includes the theoretical background of this research, followed by conceptualization and the hypotheses development that is deliberated in greater detail. Then, onward research design includes details about the data collection procedure, sampling technique, and instrumentation, followed by the data analysis outcomes outlined. Lastly, a section of discussion in which theoretical and managerial implications are described, followed by conclusions.

## Theoretical Background

### Project Management Innovation

Project managers have traditionally geared to operate under an anticipatory mindset by framing project management within codified best practices as referred to in the PMBOK^®^ Guidelines – a flagship publication of the Project Management Institute (PMI). Such standardized processes and tools have been erroneously followed by project managers as a step-by-step guideline for managing projects (Zaman et al., 2020). Adherence or compliance to such practices has been considered a reasonable assurance for minimizing project risks and managing project uncertainties. However, the unarticulated expectations from the multi-project stakeholders, especially the high-demanding project customers, have signaled a massive shift from a predictive (plan-driven) to an innovative (creativity-driven) approach to project management (Highsmith, 2009; Rincon, 2010; de Melo et al., 2021). Hence, the recent edition of PMBOK (PMBOK^®^ Guide-Sixth Edition, 2017) has introduced for the first time the agile project management practices alongside traditional approaches.

Innovation provides firms with the highest level of value creation in their project portfolios (Highsmith, 2009; Barbosa et al., 2021), while project leaders have a significant role in introducing management innovation (Volberda et al., 2013). This study underscored the definition of project management innovation which refers to “new knowledge” applications in managing projects. It includes the advancements in managerial processes that produce changes in project management strategies, structures, processes, and schemes (Birkinshaw et al., 2008; Damanpour and Aravind, 2012). Project management innovation offers techniques that are either innovative and futuristic or novel to the project-based organization (Birkinshaw et al., 2008; Maniak and Midler, 2014; Roehrich et al., 2019). Project managers can generate and apply management innovation in their unique project settings that allow similar projects to adapt to such innovative practices. Hence, the creation and adoption of “newness” to the project management approaches foster greater realization of operational and strategic goals for project-based organizations. Project management innovation may also be fundamental to a firm’s adaptation to changing environments, improving managerial processes, and achieving higher-end outcomes, especially in innovation-driven projects.

Management innovation has been widely investigated in diverse scholastic domains, including public management, corporate management, and social science, at different levels of analysis and methodological applications (Hassi, 2019; Khosravi et al., 2019). However, the examination of management innovation construct is extremely limited in a project-based environment (Thomas et al., 2013). Scholars have differentiated managerial innovation from technological innovation based on the fundamental difference in innovation outcomes. Managerial innovation refers to non-technological (i.e., administrative) innovations, whereas technological innovation includes products and procedures novelties. A large concentration of scientific research has focused on technological innovation in contrast to managerial innovation, which has recently received scholarly attention and demands more empirical investigations (Thomas et al., 2013; Khosravi et al., 2019). The deficiency in existent literature creates a lack of clarity and reliability to present a consistent set of drivers and outcomes for managerial innovation. Literature on management innovation affirmed that innovative practices have a constructive influence on organizational success through improved performance (Barbosa et al., 2021; Guertler and Sick, 2021). Past research also provides glimpses on pertinent phenomena of managerial innovation practices and innovative organizational capabilities.

Project management innovation involves the management of relationships amongst diverse project stakeholders in a highly complex social system. Management innovation in projects can achieve sustainable competitive advantage; deliver high project value and create a continuous stream of successful projects. Project management innovation fosters a broader managerial perspective focused on people, systems, and project organization (Andersen et al., 2009; Thomas et al., 2013). Managerial innovation repositions the projects in the organization by giving progressive rethought to existing project management practices (Thomas et al., 2013). The diverse nature of projects and their associated risks and uncertainties require a shift from predominant project methodologies and processes. However, prior research has completely overlooked the impact of project management innovation as it unfolds over time to achieve superior project objectives and realization of benefits for a variety of project stakeholders. The dynamics of managerial innovation in projects lead to continuous improvement, strong project management championship, and positive outcomes at an inter-organizational and intra-organizational level. Project management innovation also serves as a liberal dose of managerial creativity to the contextual variations in projects. Project management innovation brings more success to organizations through a prolonged journey of maintaining and sustaining project management efficacies. Management innovation can bring fundamental changes to the predominant belief system on existing project management practices (Thomas et al., 2013). To inspire future research, there is a need to empirically examine the relevance and impact of management innovation in a project-based environment that can offer valuable insights into understanding and facilitating project manager’s innovative capabilities (Thomas et al., 2013; Khosravi et al., 2019).

### Project Governance

Project governance is defined as a multi-layered phenomenon of governance in parent organizations that encompasses the mechanisms of interaction and relationships among projects and their multiple stakeholders (Derakhshan et al., 2019). Project governance has been considered an effective project management mechanism that heavily induces trust and engagement of project stakeholders (Müller et al., 2016). Prior research maintains a shared view on project governance as a vital system for controlling and monitoring projects, alignment of project goals with organizational strategy and protection of interests, and realization of benefits for multi-project stakeholders (Derakhshan et al., 2019; Riis et al., 2019). Research on project governance continues to intensify in a wider-organizational perspective to effectively generate and harvest greater value from managing a portfolio of projects (Riis et al., 2019).

The growing corpus of project management research has created the basis for project governance as an emerging subfield of project management. Researchers have argued that project governance can potentially become the mainstream domain of project management theory, research, and practice (Pitsis et al., 2014; Brunet, 2019). As projects have persistently aided their permanent organizations to achieve their corporate goals, hence (without exception), various enterprises are developing project governance mechanisms to capture the true value generated through projects (Lewis et al., 2002; Schoper et al., 2018; Riis et al., 2019). Multiple studies provide support for the economic and behavioral perspectives of governance in projects; however, limited research has viewed project governance from the practitioner’s perspective (Brunet, 2019). Governance as a practice in organizations and projects mobilizes the theoretical and practical contributions and dynamics in better understanding the multi-level interplay between projects and their parent organizations (Pitsis et al., 2014; Brunet, 2019). The project governance mechanism is an embedded component within the corporate (i.e., organization-wide) governance system that aims to govern a project, programs, and portfolio of projects. Hence, project governance derives strength from the corporate governance framework and established policies in organizations. Project governance mechanism is used to control and monitor the project management interface with its stakeholders, including its parent organization. This governance mechanism at the interface level ensures that projects or portfolios of projects continue to meet the organizational strategic objectives while meeting performance requirements and project goals in an organizational context (Müller et al., 2014; Simard et al., 2018; Sirisomboonsuk et al., 2018).

Project governance has been examined in two distinct streams of project management research. The first stream has viewed project governance from an intra-organizational perspective, i.e., project governance as an external factor influencing the management of projects. This implies that project governance includes the task of setting standardized governance policies that need to be complied with by all projects. However, the second stream of research considers project governance in an inter-organizational setting, such as the unique nature of projects requiring tailored project governance approaches, in contrast to the standardized practices (Ahola et al., 2014; Simard et al., 2018). In a meta-analysis of 62 studies, project governance has demonstrated a substantial role in the efficacious completion of projects (Biesenthal and Wilden, 2014). Some researchers have recommended that project governance should be directed from the high-up executive level to the project management level (Klakegg et al., 2008). A Delphi technique involving two academics and thirteen practitioners revealed that project governance does not have a formal definition. It can be described as an assortment of management systems, policies, protocols, structures and relationships that serve as a foundation of decision-taking for the progress and implementation of projects in order to achieve intended motivation for businesses or strategic purposes (Bekker and Steyn, 2007). Project governance also provides means to recognize and acquire project stakeholders’ shared interests and to effectively achieve business objectives through efficient controls and closer monitoring of projects (Sirisomboonsuk et al., 2018). Project Management Institute has defined project governance as a framework of processes and functions that guide project management activities for the creation of unique products, services, and results that help organizations to achieve their strategic, business, and operational goals (Project Management Institute [PMI], 2016). The Association for Project Management considers project governance as a subset of a corporate governance system that is directed toward project-related activities. The alignment between corporate governance (as an organizational strategy) and project governance (as an operational strategy) translates to improved organization performance (Sirisomboonsuk et al., 2018).

### High-Performance Work Practices

When organizational leaders experience an early stage success, they are most likely to focus their attention and energies on the efficient production and selling of their products and services. This apparently prudent approach can cause companies to unintentionally become one-hit wonders at the expense of enduring innovation, hence efficiently restricting their future success over time. High-performance work practices (HPWPs) are deliberated as a driving force for innovation that enables organizations to move beyond their initial success. HPWPs provide economical means of fostering innovative behaviors for sustainable success while maintaining desired efficiency levels without lagging on creativity. These practices can enhance motivation and bring meaningfulness and quality to the job, high involvement in problem-solving, and decision-taking and ultimately managerial effectiveness. Datta et al. (2005) advocated that the crux of HPWPs is to augment employees’ competence, obligation, and efficiency, enabling them to be an active part of a maintainable competitive edge.

Youndt et al. (1996) referred to HPWPs as bundled packages and was the first to theoretically define the concept. A couple of decades later, its quantitative measurement was offered in a meta-analysis conducted by Combs et al. (2006). Since then, researchers have dubbed these practices high-performance work systems, high-involvement work systems, high-commitment management practices, workplace innovations, etc., in their studies (Ogbonnaya and Nielsen, 2016). Some researchers (see Murphy et al., 2018; Ogbonnaya and Valizade, 2018) view HPWPs as taking their theoretical roots from the principles of high involvement and high commitment that are universally applicable to every business and industry. Others are of the view, that the triad framework of ability, motivation, and opportunity (AMO) forms the underpinnings for HPWPs in obtaining the desired outcomes against the organization’s extensive actions through team member’s capabilities, inspiration, and decision-taking skills (Olateju et al., 2018). Putting in either sense, these practices get together to create a multiplier effect of employees’ commitment, skills, and knowledge that reinforces each other and is greater than their impact (Dasí et al., 2021).

Following the recommendation of research, several firms have tried HPWPs to improve job satisfaction, employee’s retention, and influence organizational success (Murphy et al., 2018). Putting into a project’s perspective, these practices are yet to cement their place as project success’ determinants; however, still considered vital in creating a favorable working condition where project team members aim at regularly improving their processes. Needless to say, such practices lead to employees’ inspiration, creativeness, collaboration, proprietorship, and information sharing. The absence of these tenets could originate the emergence of unwanted outcomes such as politics, conflict, indifference, and even project failure (Hussein, 2019).

### Project Success

Project success endures to be an influential and aggressively researched topic in project management research (Ika, 2009; Müller and Turner, 2010; Musawir et al., 2017; Zaman et al., 2019a) as more and more researchers are scientifically developing lists of numerous imperative success dynamics and multi-dimensional project success standards (Carvalho and Rabechini, 2017; Zaman et al., 2019a). Traditionally a project is said to be successful if completed within predefined parameters of scope, time, and cost (Hamilton, 2004; PMI, 2017; Jitpaiboon et al., 2019). This view of projects, however, ignores several elements of the project life cycle and its context, such as contingency, complexity, constraints, and expectation of stakeholders (Hussein, 2019).

The new conception of project performance criteria takes into account efficiency, business impact, project team, and client satisfaction (Joslin and Müller, 2016; Zaman et al., 2019b). Broad set of measures like influence on clients, economy, and setting, well-organized use of means, achievement of functioning and stakeholders’ goals, project dissemination, decreased disengagements and disagreements (Shenhar and Dvir, 2007; Ika, 2009; Li and Guo, 2011; Li and Wang, 2016; Carvalho and Rabechini, 2017; Wu et al., 2017) are also considered as project success outcomes. Zwikael (2008) sees project success as project overrun, cost overrun, project performance, and client gratification. Others think of project success as the amount of transformation, i.e., modifying all or portions of the current state to an anticipated state using the products, services, or results that the project was commenced to deliver; altering the way teams work to rationalize prevailing operational mechanisms or the avenues to achieve the business objectives (Shenhar et al., 2007; Gareis, 2010; Hussein, 2019). Ika (2009) introduced the multi-dimensional assessment for measuring project success, primarily comprised of traditional measures (i.e., cost, time, and quality), but also the gratification of customers and other stakeholders. In line with Jugdev et al. (2013) suggestions, project success dynamics should be a fragment of the business’s strategic perspective and stakeholders’ expectations should be used as a guide. Building on that debate, Davis (2018) developed a survey instrument based upon the interviews of project managers and project experts that exhibited that the perception of diverse stakeholders is paramount to the final project outcome.

As this debate on defining project success continue, we rely on the conclusion drawn by Besteiro et al. (2015), that the definition of project success should be inclusive, i.e., comprising of the perspective of the stakeholder, project nature, the temporal perspective, and the organization. For this purpose, we choose Müller and Jugdev (2012) view of project success as the accomplishment of a specific set of goals and idiosyncratic measures, manifested in the achievement benchmarks and measured after the project.

### Hypotheses Development

#### Project Management Innovation and Project Success

Project management innovation (PMI) is an extension of management innovation (Mol and Birkinshaw, 2009) that aims to achieve organizational goals through the introduction of new or improved management practices and structures (Birkinshaw et al., 2008). Like management innovation, PMI seeks to increase the effectiveness of internal processes (Walker et al., 2011) so projects are completed as planned. This is the adoption of state-of-the-art organizational procedures that contribute to the performance of the project (Davies et al., 2009). Traditionally, project managers would measure success against the classical standards of completion time, allocated budgets, and quality control (Turner et al., 2010). Modern-day projects, however, are intended to meet the expectations of a number of stakeholders, requiring project managers to work more innovatively and inclusively (Albaidhani, 2019). External changes also affect the way project managers think and react. Due to shorter product lifecycles and a thinner margin of errors, companies carry the risk of losing the advantage to someone else. They are forced to continuously innovate in order to maintain existing, develop new competitive advantages, and respond proactively to changing demands (Ćirić et al., 2016; Khan et al., 2020). Similarly, every change cannot be tackled with the same techniques and requires that projects are dealt with innovation as per both currents and envisioned state requirements (Vrchota and Řehoř, 2019).

The resource-based view (Barney, 1991) framework, while providing the basis for this discussion, offers a clear path between innovation and performance (Mol and Birkinshaw, 2009). Firms, while putting their rare, valuable, and non-substitutable internal resource into action, can have a better chance to get a competitive advantage (Barney, 1991). One such venture for project managers is to consider low-cost strategies like PMI that are more difficult to replicate (Teece, 2007) and equally contribute to a longer-lasting advantage (Volberda et al., 2013). The latest research endorses the role played by PMI in the success of projects (Chen, 2014; Sergeeva and Zanello, 2018; Vrchota and Řehoř, 2019). Dulaimi et al. (2005) agreed that PMI helps managers to address challenges and tap on opportunities to ensure optimal completion. They concluded that managers of all successful projects ensured the introduction of new practices and structures throughout the life of the project. Chen (2014) also found that innovation in projects is the main determinant of project performance. He recommended that project leaders should create an environment conducive to innovation as it provides the project team with the willingness to innovate and thus leverage performance.

This is supported by Businger et al. (2020), who found that for the successful implementation of any project, project team needs to think outside of the current work flows and processes and bring necessary modifications throughout. Jissink et al. (2019), advised project managers to inspire innovation with the help of forward-looking activities such as assessing industry trends, competitors’ responses, technological advancement, and utilization of relevant knowledge. Unnecessary to say, the project way of managing innovation is the competitive advantage for every organization these days (Vrchota and Řehoř, 2019).

H_1_: Project management innovation has a significant and positive effect on project success.

#### Moderating Effect of Project Governance

Project governance has emerged as a topic of interest for researchers these days (Musawir et al., 2020). It refers to the organizational governance of a project, i.e., the protocols used by the project managers to control, direct, and guide projects to meet their objectives (McGrath and Whitty, 2015). The definition, communication, and documentation of reliable and repeatable project practices that are believed to be essential for project success come under the domain of project governance (PMI, 2013, p. 34). A growing consensus reveals that for projects and project-based organizations to be effective, suitable project governance measures are paramount (Müller et al., 2015; Ahola et al., 2014). This conception takes its roots from corporate governance literature, which reveals that optimized governance mechanisms result in weaker agency problems, hence higher corporate performance (Hart, 1995; John and Senbet, 1998; Ozkan, 2007; Hirschey et al., 2009). Unlike traditional project success criteria developed on operational and tangible measures (time, cost, and financial return), project governance views success as the strategic, long-term, and social impact of the project’s outcome (Sanderson, 2012; Samset and Volden, 2016). For this purpose, research stresses project governance practices to provide the project teams with the autonomy, authority, and expertise to create tangible value for their client (Lappi et al., 2018). Joslin and Müller (2016) assert project governance is an embedded part of project’s context that influences both project’s methodology and outcome.

Burns and Stalker (1961) portrayed context as the most important variable triggering a change and stressed the organizations’ adjustment in response to changes in the outer world. This view is supported by contingency theory (Fiedler, 1964) which writes off the existence of a universal approach to making organizational decisions. The framework which is widely applied to examine the suitability of various governance structures in different project contexts (Musawir et al., 2020) brings into play the existing internal and external environmental factors for consideration toward optimized project management. This is why Shenhar and Dvir (2007), Rolstadås et al. (2014), and Hussein (2019) advised project managers to scan and understand the project context before deciding on how to manage the project.

Referring to Narayanan and Narasimhan (2014), the empirical support for project governance to moderate between PMI and PS does come from the literature as a number of organizational studies propose governance as circumstantial structure and employ it as a moderating factor. For example, Bekker and Steyn (2007) supported the role of governance principles in project success; Müller and Martinsuo (2015) asserted that project governance moderates the association of relational norms among clients and suppliers to jointly impact project’s success; Müller et al. (2017) found it to be significantly moderating between the governance of projects and project success. Similarly, the finding of Wang and Chen (2006) about project governance as an important ingredient for IT projects strengthens this view and guides us to hypothesize that:

H_2_: Project governance positively moderates project management innovation and project success.

#### Moderating Effect of High-Performance Work Practices

The focus of human resource management (HRM) has changed over time from traditional employment known for its limited employee participation; to a more engaging process that considers employees as a resource fully capable of giving the company a competitive edge. This shift has opened the doors for HPWPs in exploiting the human resource’s potential for competitive advantage. Research conducted in the last couple of decades has evidenced the worth of HPWPs for organizational performance in a number of industries and geographies (Kloutsiniotis and Mihail, 2018), where HPWPs have been consistently acknowledged as productivity-enhancers. An optimized package of HPWPs enables managers to strategically guide employees’ performance toward corporate performance (Obeidat, 2017). This connection is understood through the ability-motivation-opportunity (AMO) framework (Appelbaum et al., 2000), which asserts that skilled worker performs better and if motivated, actively seek opportunities for improvement. HPWPs hugely affect employees’ knowledge, abilities, and make available the opportunities to employees so they make use of them (Armstrong et al., 2010). Garcia and Tomas (2016) argued that HPWPs have the ability-augmenting prospective for updated knowledge, expertise, and pre-dispositions; motivation-augmenting prospective for desired behavior so to improve employee contribution in achieving overall goals; and opportunity-improving prospective for knowledge exchange, cross-functional communication, participative decision-taking and training transmission prospects.

Kang et al. (2018) stressed developing employees’ competence through modern techniques to address the ever-increasing demands of various industries. On the project side, the effect of HPWPs, i.e., training, teamwork, constant feedback, recognition, and rewards on project success was analyzed by Olateju et al. (2018), who found a strategic linkage between the two. The study revealed that project dynamics demand HPWPs that can help project managers in making necessary modifications to project scope, increase competencies, ensure project team’s motivation, and facilitate participative decision taking. Such interventions function as facilitators for project-orientated organizations to ensure project success by developing a knowledgeable labor force, building project teams, growing employee engagement, and participation, and promoting knowledge exchange (Zaman, 2020). Project success probability is increased as HPWPs foster favorable work perceptions and spirited organizational situations to meet established performance standards (Wickramasinghe and Liyanage, 2013; Khan and Rasheed, 2015).

High-performance work practices would increase project management effectiveness if project members completely understood all the project stages required to complete the tasks (Jitpaiboon et al., 2019). Bhatti et al. (2021) indicated that at the individual project level, innovation is higher when employee abilities and motivation are the focus of HPWPs (Hafeez et al., 2020). That is why project-based organizations are innovative as skilled and prepared employees are entrusted to commence exhaustive knowledge undertakings (Theodorou et al., 2019). Hence preparation, motivation and impetus form the foundation for better performance at the project level, while a dearth of trained workers is the most restraining element (Dasí et al., 2021). However, HPWPs can foster prospects to upgrade skills, knowledge sharing, use of shared learning, and novel ideas for project’s effectiveness (Zavadskas et al., 2014; Ogbonnaya and Valizade, 2018; Olateju et al., 2018). The literature does link HPWPs to an improved project and employee performance in project-based organizations (Wickramasinghe and Liyanage, 2013; Popaitoon and Siengthai, 2014).

H_3_: HPWPs positively moderate project management innovation and project success.

**FIGURE 1:**
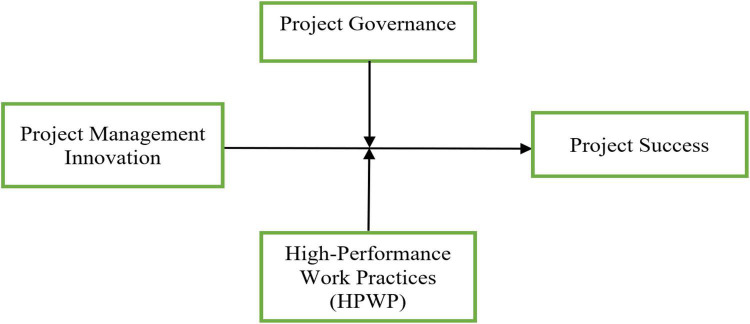
Conceptual model.

Figure 1 illustrates our research framework that proposes the following hypothesized relationships: (1) the direct effect of project management innovation on project success; (2) the moderating effect of project governance; and (3) the moderating effect of high-performance work practices.

## Methods

### Sampling and Procedure

The deductive-quantitative approach in combination with the prevailing method of a cross-sectional survey in project management research was used to assess the proposed relationships (Joslin and Müller, 2016; Musawir et al., 2017). The sampling frame consisted of project professionals: team members, coordinators, and consultants working in the software development sector of Pakistan. These are the people who sense, experience, and witness the outcomes of ICT projects (Zhang et al., 2018). A target of a minimum of 280 (40*7) valid responses was set as per the recommendation of Hair et al. (1995), for which the researchers floated 600 soft copies through direct email communications, and by posting questionnaire using Google-form online survey link through several digital platforms such as social networking service (SNS), LinkedIn, Facebook, and WhatsApp. The respondents were taken into confidence by ensuring that the confidentially of their responses would be maintained and only used for academic purposes. Upon the examination of 427 returned copies, 315 responses were found valid and considered for analysis setting the effective response rate as 52%, slightly higher than the average response rate of 49% in Pakistan (Saeed, 2016).

### Measures

The study used modified scales to measure the current study variables that were employed in earlier studies. Sufficient care was taken to maintain the content validity with the help of careful operationalization of the constructs and scale selection. As per the recommendation of Cooper and Schindler (2011), a panel of experts comprising university professors and project professionals were also engaged in examining the instrument. Five-point Likert scale ranging from “strongly disagree = 1 to strongly agree = 5” was used to capture responses. Confirmatory factor analysis (CFA) was run to confirm the adapted measurement scale passed the criteria for reliability and validity.

#### Project Management Innovation

Currently, the scale to measure project management innovation in an organizational context does not exist, thus the items for these variables were compiled based on, J Nieves and Segarra-Ciprés (2015) and Nieves (2016). This resulted in a five-item scale that broadly covered what project managers do, how they do it, as well as the organizational setup in which the work is performed. Specifically, it measured the magnitude and effectiveness of changes in the areas of decision making, team responsibilities, managing stakeholder relationships, and management effectiveness at the project level.

#### Project Governance

The project governance questions were taken from Musawir et al. (2017), who initially developed this scale on the principles of corporate governance. The idea was to adjust the mechanism of governance to the project level. The opinion of participants was obtained using the 9-item long scale that targeted the main governance-related issues in a project, such as the establishment of responsibility and accountability, working discipline, role and goal clarity, risk management, and adequate disclosure about the project at hand.

#### High-Performance Work Practices

The HPWPs scale was derived from Olateju et al. (2018), who prepared the same to examine the effect of these practices on project success in various industries like construction, information communication technology, and transportation. The scale consists of core HPWPs, i.e., training and development (two items), rewards (two items), teamwork (6 items), recognition (three items), and continuous feedback (two items). The 15-item scale has exhibited sufficient reliability in studying HPWPs in the domain of project management (Olateju et al., 2018).

#### Project Success

For project success, the 11-item scale was taken from Musawir et al. (2017), which was developed by Serra and Kunc (2015) and Zwikael and Smyrk (2015) and is based on the triple-test performance framework. Five items measured project management success, three items measured project ownership success, and three items measured project investment success. Broadly, the scale was intended to test the ability of the project to deliver its intended output, support for the business case, and achievement of ROI (Zaman et al., 2022b).

### Data Analysis

To empirically test the direct impact of project management innovation on success and indirectly under the moderating conditions of project governance and HPWPs, the structural equation modeling (SEM) technique of AMOS was employed. SEM is a well-known technique to statistically measure and study a variety of relationships in complicated models (Hair et al., 2017; Zaman et al., 2019b). Courtesy of its easy-to-understand path analysis, SEM lets the researchers examine a combination of interrelated research questions systematically and inclusively (Anderson and Gerbing, 1988). An increasing number of studies in the areas of supply chain management, human resource management, marketing, tourism, accounting, and strategic management are employing SEM to good effect (Hair et al., 2017; Zaman et al., 2021a).

## Results

### Correlations

Prior to hypothesis testing, we analyzed correlations between the measured constructs. These results are presented in Table 1, along with descriptive statistics. The correlation among all the constructs is on the higher side ranging from 0.571 to 0.706, which reveals a close association between them. It can be observed that all the alpha reliabilities fall within the acceptable range and the bivariate correlations move in the right direction. Results are statistically significant at 0.01.

**TABLE 1:** Descriptive and correlation analysis.

Variables	M	SD	α	PMI	HPWP	PG	PS
PMI	4.0244	0.740	0.88	1			
HPWP	3.9266	0.761	0.91	0.571*	1		
PG	3.8051	0.629	0.81	0.612*	0.684*	1	
PS	3.9674	0.730	0.94	0.679*	0.706*	0.659*	1

*Correlation is significant at the 0.01 level (two-tailed), PMI, project management innovation; HPWP, high-performance work practices; PG, Project performance; PS, project success.

### Model Fitness

After correlation analysis, the models were assessed for their goodness with the help of several indices. These included chi-square test, goodness of fit index (GFI), incremental fit index (IFI), comparative fit index (CFI), Tucker and Lewis (1973) index (TLI), root-mean-square error of approximation (RMSEA), and the standardized root-mean-square residual (SRMR). The Chi-squared (*χ2*) value is 1595.516 and *χ2*/*df* value is 2.57, which is lesser than the maximum value of 3. Other indices also passed comfortably the recommended values of model fitness as shown in Table 2. The overall model’s fit is thus extremely good and fulfills the validity requirement.

**TABLE 2:** Measurement model fit indices.

Model	*x* ^2^	*x*^2^/df	GFI	IFI	CFI	TLI	RMSEA	SRMR
Recommended Values	−	<3.0	>0.90	>0.90	>0.90	>0.90	<0.60	<0.08
Measurement Model	1595.516	2.57	0.92	0.91	0.91	0.90	0.060	0.053
Structural Model	2081.299	3.34	0.98	0.97	0.96	0.95	0.034	0.045

### Validity

**FIGURE 2:**
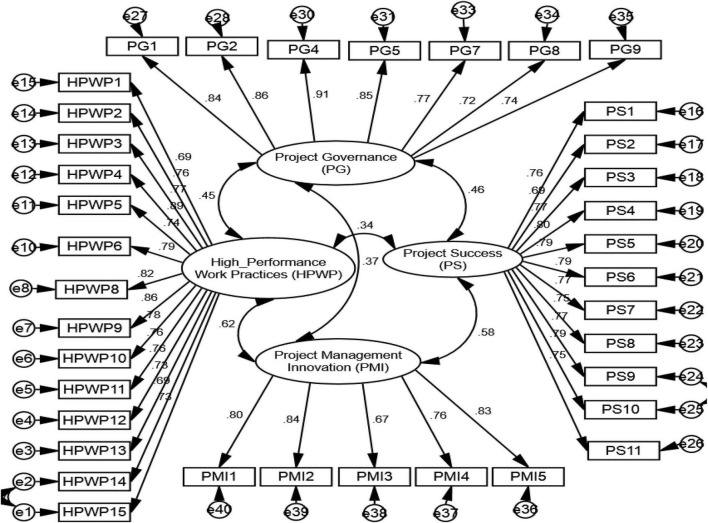
Measurement model.

The convergent and discriminant validity of the scales were achieved through Cronbach’s alpha coefficients, average variance extracted (AVE), and factor loadings using CFA. The convergent validity confirms the degree of agreement among numerous items or indicators of the same construct, while discriminant validity tests how far a measure moves away from another measure whose underlying construct is theoretically not relevant. Cronbach’s alpha coefficients of each dimension comfortably surpassed the suggested value of 0.70 (as shown in Table 3), largely regarded as sufficient for testing reliability (Kline, 2015). Similarly, the AVE values of all variables exceeded the benchmark value, i.e., 0.5 (Hair et al., 2017), as shown in Table 3. These values confirm the unidimensionality of the composites and the authenticity of convergent validity. After all, CFA was conducted to look for items with minimum loading required, which resulted in the removal of 3 items, i.e., one from the HPWPs scale and two from the PG scale (as shown in Table 4 and Figure 2).

**TABLE 3:** Convergent and discriminant validity.

Variables	CR	AVE	PMI	HPWP	PG	PS
PMI	0.886	0.612	(0.782)			
HPWP	0.953	0.593	0.62	(0.771)		
PG	0.932	0.664	0.37	0.45	(0.815)	
PS	0.940	0.588	0.58	0.34	0.46	(0.767)

*AVE, average variance extracted; CR, composite reliability. Values in parentheses represent the square root of AVE.*

**TABLE 4:** Confirmatory factor analysis: standardized loading.

Items	PMI	HPWP	PG	PS
PMI1	0.80			
PMI2	0.84			
PMI3	0.67			
PMI4	0.76			
PMI5	0.83			
HPWP1		0.69		
HPWP2		0.76		
HPWP3		0.77		
HPWP4		0.89		
HPWP5		0.74		
HPWP6		0.79		
HPWP7		0.41*		
HPWP8		0.82		
HPWP9		0.86		
HPWP10		0.78		
HPWP11		0.76		
HPWP12		0.75		
HPWP13		0.73		
HPWP14		0.69		
HPWP15		0.73		
PG1			0.84	
PG2			0.86	
PG3			0.42*	
PG4			0.91	
PG5			0.85	
PG6			0.56*	
PG7			0.77	
PG8			0.72	
PG9			0.74	
PS1				0.76
PS2				0.69
PS3				0.77
PS4				0.80
PS5				0.79
PS6				0.79
PS7				0.77
PS8				0.75
PS9				0.77
PS10				0.79
PS11				0.75

*Items deleted (03 items were deleted to achieve model fitness HPWP7, PG3, and PG6).

### Structural Model for Hypothesis Testing

**FIGURE 3:**
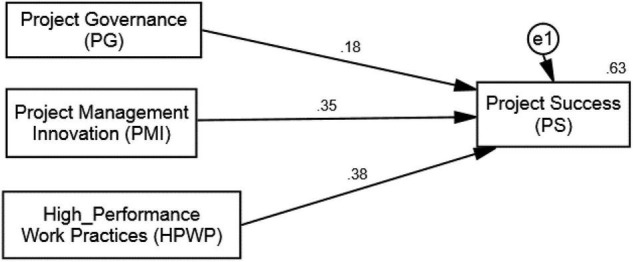
Structural model 1 (direct paths without moderators).

**FIGURE 4:**
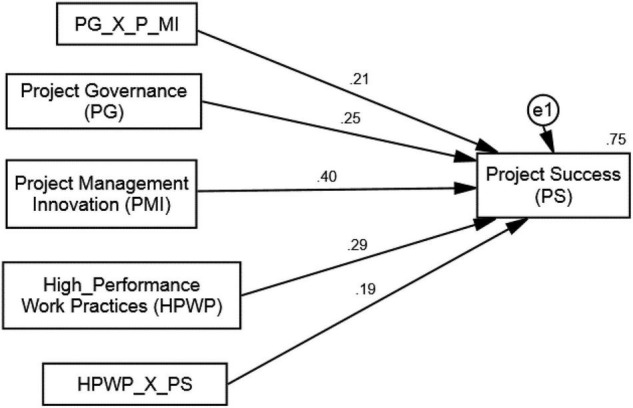
Structural model 2 (with two moderators).

The results of the SEM analysis are presented in Table 5, with PS as the dependent variable (Ahmad et al., 2020). Two models were examined in this analysis, i.e., Structural Model 1 and Structural Model 2. Structural Model 1 reflects the direct paths, while Structural Model 2 includes the paths influenced by the moderators. Model 1 confirms the hypothesized positive relationship between PMI and PS and thus validates the hypothesis 1 (β = 0.35; *t* = 9.046; *p* < 0.01). This proposes that the adoption of high-level innovation practices by project managers leads to greater chances of success of the project at hand. The 63% variation (*R*^2^ = 0.63) caused by the constructs of Model 1 in the dependent variable further solidifies this result. The graphical diagram of this analysis appears in Figure 3. Hypothesis 2 (β = 0.21; *t* = 10.16; *p* < 0.01) and hypothesis 3 (β = 0.19; *t* = 7.90; *p* < 0.01) are also accepted. The Structural Model 2 explains the indirect relationships and the results suggest that both PG and HPWPs play significant moderating roles which strengthen the relationship between PMI and PS (Zaman et al., 2022b). It can be observed that with the inclusion of the moderators in the model, its explanatory power increased from 63 (*R*^2^ = 0.63) to 75% (*R*^2^ = 0.75). With improved PG and HPWPs, project managers achieve desired project outcomes with the help of PMI as a determinant. The results of moderation are given in Table 5 (indirect paths) and Figure 4.

**TABLE 5:** CB-SEM estimations for hypothesis testing.

Model 1: Direct paths	β	*t*-Value	*P*	*R* ^2^	Hypothesis
PG → PS	0.18	4.269	<0.01	0.63	
HPWP → PS	0.38	9.161	<0.01		
PMI → PS	0.35	9.046	<0.01		H1 Supported
**Model 2: Moderators**					
PMI → PS	0.40	12.61	<0.01	0.75	
PG-X-MPMI → PS	0.21	10.16	<0.01		H2 Supported
HPWP-X-MPMI → PS	0.19	7.90	<0.01		H3 Supported

*PMI, project management innovation; HPWP, high-performance work practices; PG, project performance; PS, project success.*

## Discussion

### Interpretation of the Study Findings

The current study confirmed project management innovation as an important determinant of project success. The results show that project managers can increase the chances of project success when they bring new and improved approaches to the management of the project. These results are in line with RBV, which sees PMI as one of the rare, valuable, durable, and non-substitutable sources of advantage that can lead to their desired outcome (Camisón and Villar-López, 2014). This encouraging result expands the scope of innovation from that of technological and management innovation to the arena of project management (Allahar, 2019). An increasing number of studies endorse the positive impact of project management innovation on project success, for example, Alawamleh et al. (2020) advised project managers to encourage innovation irrespective of the nature of the project. They argued that employing innovative approaches to thinking and doing by project managers helps the firm attain a competitive advantage and promote its market position. Researchers are also showing interest in applying the concept of innovation to the various phases of a project; for example, Allahar (2019) viewed its value in the project planning phase. The study suggested that project managers should employ design thinking, system analysis, and milestone charting to enhance the quality of the project in the planning phase and obtain desired outcomes at the end. Another study by Keshtiaray and Vazifehdust (2020), found that the use of innovation in project management has a positive and significant effect on reducing environmental pollution. Severo et al. (2020) debated that innovation is at the heart of project management that adds to the sustainability of the project at hand and seeks to achieve a competitive advantage. Project management innovation may also be fundamental to firm’s adaptation to changing environments, improving managerial processes, and achieving higher-end outcomes, especially in innovation-driven projects.

The current study also found project governance as a key moderator, just like it was found in previous studies (Müller et al., 2017) involving project success. It is interpreted that the relationship between project management innovation and project success is strengthened when certain project governance standards are set in place. After analyzing four “well-managed but failed” projects, Kiselev et al. (2020) encouraged executives to set project governance as a key priority when implementing their project strategies to ensure project success. The importance of project governance for project performance will only grow as the pressure on sustainability mounts (Sankaran et al., 2021). Sirisomboonsuk et al. (2018) witnessed that project governance had a positive influence on the performance of the project. Project governance becomes even more critical when the project at hand is of significance, bears a greater amount of risk, counters performance gaps, and needs to be readjusted to a change in managerial context or strategic preferences (Crawford et al., 2008). However, the PG framework implementation has to ensure a rational balancing act when endorsing structures and practices Musawir et al. (2020). It includes choosing a suitable governance system keeping in view the project’s characteristics as well as the environment where it operates. Thus it is unwise to apply a single project governance system universally across all projects. Contingency theory suggests managers should try to establish a balance between the organizational attributes and the context where it operates to achieve higher performance levels. For this study, it translates into the interplay of project management innovation, project governance, and HPWPs that affect each other to find their balance in the shape of project success.

H3 is upheld, which cements our argument that HPWPs are pretty much relevant to projects because of several reasons: project members’ job responsibilities are changing at a rapid pace due to environmental fluctuations; team performance accountability; and lateral coordination based on common goals with lesser status differences (Gollan et al., 2005). Earlier studies did find a strong positive effect of HPWPs on PS in many sectors (Olateju et al., 2018). For example, Zaman (2020) examined the role of HPWPs in influencing the success of mega projects. The study stressed that the integrated and synergistic nature of HPWPs can generate more instances of viable and successful projects. As recommended by the AMO framework, studies conducted recently have supported the development of employees’ abilities and skills through innovative approaches to address the changing demands of different industries (Kang et al., 2018). Bhatti et al. (2021), concluded that innovation performances increase when HPWPs are aimed at establishing job clarity, and increasing employee abilities and commitment. This association was also observed by Wickramasinghe and Liyanage (2013) in their study consisting of software developers who found that the individual performance of project members is positively affected by the HPWPs. The current study goes further and specifies high-performance work practices, including training and development, recognition and reward, communication, flexibility, participation, appreciation, and performance feedback. These practices increase employees’ ability and stimulate their intent to innovate; hence improving the performance of the projects ultimately.

### Theoretical Implications

A plethora of studies are available which discuss innovation project management, open innovation, and using projects as innovation tools, however, project management innovation as a concept is comparatively fresh and the current study is the first to empirically test its impact on project success individually and jointly with project governance and HPWPs. Theoretically, this research sets the tone for further research endeavors involving project management and project performance. The effective addition of PG and HPWPs to the framework has also extended the debate of finding suitable important factors that facilitate project performance. In this standard, this research advances the understanding of the role of the enabling factors such as project governance and HPWP. The results demonstrated that project governance and HPWP function as influential factors toward the adoption of innovation management practices. Theoretically, the success of the project innovation management is highly reliant on the better execution of the project. From the organizational perspective, project governance is a valuable tool that can be applied to decrease the resistance of the stakeholders. Previous literature suggests that project governance is a central function mechanism that outlines managerial actions for endorsing influences on the project’s success. The results of this research shed light on the critical importance of project governance which is how it aids the management to convey timely requirements as well as provides a platform for resolving issues. Likewise, the moderating implications of the HPWPs have established that increasing project success is possible through generating circumstances that support employees’ involvement. The greater intensity of motivated employees with sophisticated required skills through HPWP’s can increase the chances of timely accomplishment of project objectives.

### Managerial Implications

In practical terms, the current study suggests that project managers should aggressively weigh value-adding ideas throughout the life of the project in pursuit of the desired outcome. Project leaders need to encourage the innovation process by building dynamic capabilities through the combination of tasks and approaches, inculcating a culture of problem-solving, setting benchmarks from other sectors, and demonstrating serial management innovation. In favor of HPWPs, the study recommends that project leaders should update the knowledge of employees and staff members necessary for them to bring new and innovative ideas to the project. One way to achieve this is to let the employees participate in relevant seminars, workshops, and training that discuss innovation in project management in specific and business in general. Managers should also realize that projects are not undertaken in isolation but alignment with the larger business objectives. Thus they should adopt a suitable PG mechanism to complete the project at hand as well as contribute toward the overall organizational goal achievement without experiencing a conflict of interest. Adopting a fitting PG structure characterized by transparency, adequate disclosure, accountability, risk management, and role clarity may make the project manager’s job easier in achieving the project and organizational objectives. Therefore, organizations consider adopting the HPWPs and clear execution of the projects through crafting an environment that diminishes the innovation resistance among the stakeholders. Thereby, the inclusion of extensive training is needed for preparing the organizational stakeholders to ensure the smooth adoption of project innovation practices. This involves a variety of structural procedural initiatives such as recognition and reward, to encourage the employees to vigorously engage to accomplish the project goals. Similarly, the development and management of the informational channels are also critical for ensuring the participation of the stakeholders through the availability of feedback channels. These steps can encourage the employees to innovate and ultimately achieve successful outcomes in their projects.

### Limitations and Future Research

The current study throws PMI as a fresh concept that demands further understanding. Future research should employ more rigorous methodological approaches such as qualitative analysis, operationalization, and scale development to get hold of the new concept. Second, the current study does reveal the positive perception of project professionals about PMI in relation to PS; however, innovation is never a walk in the park. Thus it would be interesting to inquire about the possible obstacles to innovation, especially when the project managers are caught up in deadlines. Third, the current framework was tested by involving project professionals from physical teams making the study findings less generalizable to teams that are virtually connected. This becomes an ideal venue to test the same model and evaluate the interplay of PMI, PG, HPWPs, and PS in virtual settings. Fourth, it should be noted that there is no universal PG mechanism; however, the researchers can agree on a set of golden governance rules which can generate results in a variety of circumstances. That golden set of governance needs to be explored. The current study is limited to the software development sector. Therefore, the generalizability is restricted. It could be replicated in other project areas such as construction, IT, and mega projects. Finally, the study used a cross-sectional mode of inquiry; however, PMI cannot be measured directly through performance as its benefits are not immediate and less evident (Wong, 2013). Thus a longitudinal approach will deem fit to understand the long-term impact of PMI on PS. Future studies should also explore how team voice and/or team silence can serve as a potential mediator(s) between project management innovation and project success (Zaman et al., 2022c), also how the political and social skills of project managers can interplay as potential moderators in these relationships (Zaman et al., 2019b).

## Conclusion

Modern businesses take their projects seriously and link the project success to the firm success. The renewed focus of contemporary organizations has sparked an interest of both researchers and practitioners to look for and implement improved mechanisms and methodologies for project performance. Yet, the success rate statistic across the globe is not satisfactory, as reported by many researchers. The current study endeavors to find an answer to the question of how to improve project performance by closely examining the interplay of project management innovation, project governance, HPWPs, and project success. An adapted scale was used to test this set of hypotheses with the help of data collected from project professionals having sufficient project management experience in the software development sector of Pakistan. All three hypotheses are validated concluding that project management innovation can lead to project success. The results also suggested that both project governance and HPWPs have a positive moderating impact on the relationship between project management innovation and project success. These results provided evidence to project management professionals to consider PMI as an important PS determinant while both PG and HPWPs as PS enablers. All three can be used to bring about significant improvement as far as the project outcomes are concerned. In the end, the study suggests new venues of research to build on the debate concerning project effectiveness, governance, and adoption of best HR practices.

## Data Availability Statement

The raw data supporting the conclusion of this article will be made available by the authors, without undue reservation.

## Author Contributions

UZ, MK, SR, and PF contributed to conceptualization, methodology, software, validation, investigation, data curation, writing—original draft preparation, writing—review and editing, visualization, and supervision. UZ, MK, and SR contributed to formal analysis. UZ, MK, and PF contributed to resources and project administration. All authors contributed to the article and approved the submitted version.

## Conflict of Interest

The authors declare that the research was conducted in the absence of any commercial or financial relationships that could be construed as a potential conflict of interest.

## Publisher’s Note

All claims expressed in this article are solely those of the authors and do not necessarily represent those of their affiliated organizations, or those of the publisher, the editors and the reviewers. Any product that may be evaluated in this article, or claim that may be made by its manufacturer, is not guaranteed or endorsed by the publisher.
